# Detection of EEG-resting state independent networks by eLORETA-ICA method

**DOI:** 10.3389/fnhum.2015.00031

**Published:** 2015-02-10

**Authors:** Yasunori Aoki, Ryouhei Ishii, Roberto D. Pascual-Marqui, Leonides Canuet, Shunichiro Ikeda, Masahiro Hata, Kaoru Imajo, Haruyasu Matsuzaki, Toshimitsu Musha, Takashi Asada, Masao Iwase, Masatoshi Takeda

**Affiliations:** ^1^Department of Psychiatry, Osaka University Graduate School of MedicineOsaka, Japan; ^2^The KEY Institute for Brain-Mind Research, University Hospital of PsychiatryZurich, Switzerland; ^3^Department of Neuropsychiatry, Kansai Medical UniversityOsaka, Japan; ^4^UCM-UPM Centre for Biomedical Technology, Department of Cognitive and Computational Neuroscience, Complutense University of MadridMadrid, Spain; ^5^Nihon KohdenShinjuku, Tokyo, Japan; ^6^Brain Functions Laboratory IncorporatedYokohama, Japan; ^7^Department of Neuropsychiatry, Institute of Clinical Medicine, University of TsukubaTsukuba, Japan

**Keywords:** eLORETA-ICA, LORETA, resting state network, independent component analysis, ICA, EEG

## Abstract

Recent functional magnetic resonance imaging (fMRI) studies have shown that functional networks can be extracted even from resting state data, the so called “Resting State independent Networks” (RS-independent-Ns) by applying independent component analysis (ICA). However, compared to fMRI, electroencephalography (EEG) and magnetoencephalography (MEG) have much higher temporal resolution and provide a direct estimation of cortical activity. To date, MEG studies have applied ICA for separate frequency bands only, disregarding cross-frequency couplings. In this study, we aimed to detect EEG-RS-independent-Ns and their interactions in all frequency bands. We applied exact low resolution brain electromagnetic tomography-ICA (eLORETA-ICA) to resting-state EEG data in 80 healthy subjects using five frequency bands (delta, theta, alpha, beta and gamma band) and found five RS-independent-Ns in alpha, beta and gamma frequency bands. Next, taking into account previous neuroimaging findings, five RS-independent-Ns were identified: (1) the visual network in alpha frequency band, (2) dual-process of visual perception network, characterized by a negative correlation between the right ventral visual pathway (VVP) in alpha and beta frequency bands and left posterior dorsal visual pathway (DVP) in alpha frequency band, (3) self-referential processing network, characterized by a negative correlation between the medial prefrontal cortex (mPFC) in beta frequency band and right temporoparietal junction (TPJ) in alpha frequency band, (4) dual-process of memory perception network, functionally related to a negative correlation between the left VVP and the precuneus in alpha frequency band; and (5) sensorimotor network in beta and gamma frequency bands. We selected eLORETA-ICA which has many advantages over the other network visualization methods and overall findings indicate that eLORETA-ICA with EEG data can identify five RS-independent-Ns in their intrinsic frequency bands, and correct correlations within RS-independent-Ns.

## Introduction

The brain intrinsically interacts between distant regions, building cortical networks during motor and cognitive tasks. Interestingly, one network enhances its activity in no-task resting state. In particular, the so called default mode network (DMN) is known to be active during resting and attenuates during task performance. However, recent findings suggest that the DMN is also involved in internally focused processes such as self-referential thoughts, envisioning one’s future and autobiographical memory retrieval (Raichle et al., 2001; Buckner et al., 2008). Furthermore, it has been reported that several cortical networks cooperate with each other positively or negatively during performance of complex cognitive tasks (Spreng and Schacter, 2012). These functional networks have been investigated by lesional and anatomical studies and during functional tasks with functional magnetic resonance imaging (fMRI), which measures regional cerebral blood flow (rCBF) changes. However, one mathematical method called independent component analysis (ICA) have received growing attention (Bell and Sejnowski, 1995; Hyvärinen and Oja, 2000). ICA is a mathematical decomposing method which separates mixture of signals like electroencephalography (EEG), magnetoencephalography (MEG) and fMRI data into a set of statistical independent components (ICs) that are artifact signals and physiological signals. In addition, it should be noted that using ICA these task positive or negative functional networks can be extracted from “resting state” fMRI data and MEG data (Beckmann et al., 2005; Allen et al., 2011; Brookes et al., 2011). These led to the concept of “Resting State independent Network” (RS-independent-N). Also, there are some other methods used for the discovery of interactions in the brain which are seed-based correlation analyses. These analyses has extracted Resting State correlated Networks (RS-correlated-Ns) from resting state fMRI data or MEG data (Biswal et al., 1995; Vincent et al., 2008; Brookes et al., 2011; Raichle, 2011; Hipp et al., 2012). In this way, ICA and seed-based correlation analyses with fMRI data has identified several RS-independent-Ns and RS-correlated-Ns, including the basal ganglia network, auditory network, sensorimotor network, visual network, DMN, ventral and dorsal visual pathway (VVP and DVP), and the frontal network (Biswal et al., 1995; Allen et al., 2011; Joel et al., 2011; Raichle, 2011; Meyer et al., 2013). However, correlation analysis has a problem of an implicit assumption of Gaussianity of the signal where fMRI signals are approximately Gaussian (Hlinka et al., 2011) but EEG and MEG signals are non-Gaussian (Stam, 2005). Thus, RS-correlated-Ns derived from correlation analysis of EEG and MEG data are not independent with each other in a precise sense because of non- Gaussianity of EEG and MEG data (Hyvärinen and Oja, 2000; Stam, 2005). In addition, correlation analyses emphasize the special role of some pre-selected brain region. However, unlike the seed-based methods, ICA is appropriate for the discovery of distributed networks, giving equal importance to all brain voxels (Joel et al., 2011). Furthermore, ICA can remove artifacts such as electromyogram or base line shift by separating out artifact components (Custo et al., 2014).

Unlike fMRI, which measures hemodynamic changes that occur in response to cortical activity, neurophysiological techniques, such as EEG and MEG measure cortical electrical/magnetic activity directly and noninvasively with a high temporal resolution (1–2 ms) (Canuet et al., 2011). Thus, EEG has been widely used in clinical practice to support clinical diagnosis and management of neuropsychiatric diseases such as epilepsy, disturbance of consciousness and dementia, and also in neuroscience to investigate cortical electrical activities and functions (Ishii et al., 1999; Canuet et al., 2011; Kurimoto et al., 2012; Aoki et al., 2013a,b).

Recent findings of EEG and MEG analyses indicate that electromagnetic oscillatory activity of the functional networks varies its frequency from lower sensory areas to higher-order control areas. For instance, intra-cortical investigations using depth electrodes with syllable auditory task reported that cortical electrical activity of auditory area changed from evoked activity (phase-locked to the stimulus) to induced activity (non-phase-locked to the stimulus) and also its frequency changed from theta and low gamma to beta and high gamma, as activity shifted from primary auditory cortices to associative auditory cortex (Morillon et al., 2012). Another MEG study using a visuospatial attentional task found that the cortical electrical activity of the DVP changed from alpha evoked activity to beta induced activity as it shifted from early visual areas to prefrontal control areas (Siegel et al., 2008). And recent fMRI and MEG studies using decomposing methods have repeatedly shown that these functional networks can also be seen during resting state with changing its power of activity (Smith et al., 2009; Grady et al., 2010; Brookes et al., 2011). From these accumulating evidences, we can assume that RS-independent-Ns are associated with several frequency bands of electromagnetic activity depending on the function subserved by the different cortical regions. In support of this notion, a simultaneous fMRI and EEG study showed that blood oxygenation level dependent (BOLD) signals of RS-independent-Ns correlated with EEG waveforms in several frequency bands (Mantini et al., 2007). In addition, Jonmohamadi et al. (2014) and Mantini et al. (2011) showed that ICA decomposition of EEG and MEG data becomes more correct in localization and more robust to artifacts when applied after source reconstruction. Taken together, in order to visualize RS-independent-Ns across several frequency bands, we consider appropriate to apply ICA to cortical electrical activity reconstructed from EEG or MEG data, analyzing all frequency bands. To our knowledge, there is one previous EEG-RS-independent-N study. However, ICA was applied to scalp recorded EEG data in the time domain, followed by a second step using a sLORETA source reconstruction on the ICA-scalp topographies; in contrast, we apply ICA directly to the reconstructed cortical electrical activity by eLORETA in the frequency domain. And the results of cortical electrical distributions of ICs were rather different from known functional networks (Chen et al., 2013). Also there is a few previous MEG-RS-independent-N studies. In their studies, ICA was applied to cortical electrical activity reconstructed from MEG data, however, in separate frequency bands, disregarding possible cross-frequency coupling. Furthermore, sample sizes of these studies were small (Brookes et al., 2011, 2012; Luckhoo et al., 2012).

Also, ICA of EEG data has been widely used for various purposes, such as artifact rejection by separating out artifact components (Custo et al., 2014) and examination of the EEG resting states (infra-slow EEG fluctuations and EEG microstates). For instance, Hiltunen et al. (2014), found correlations between the filtered ICA time series (using ultra-low frequencies) of the EEG with BOLD time series in specific fMRI RS-independent-Ns. And Yuan et al. (2012), performing ICA on EEG microstates to decompose into ICs (independent microstates), found that each fMRI RS-independent-N was characterized by one to a combination of several independent microstates.

Exact low resolution brain electromagnetic tomography (eLORETA) is a linear inverse solution method that can reconstruct cortical electrical activity with correct localization from the scalp EEG data (Pascual-Marqui et al., 2011; Aoki et al., 2013a). The implementation of ICA in the eLORETA software with EEG data allows for decomposition of cortical electrical activity which is non-Gaussian into ICs in different frequency bands (Pascual-Marqui and Biscay-Lirio, 2011). Other decomposing methods (e.g., principal component analysis or correlation analysis) with EEG data cannot strictly to do so (Bell and Sejnowski, 1997; Hyvärinen and Oja, 2000; Mantini et al., 2011). Furthermore, electromagnetic tomography-ICA (eLORETA-ICA) uses all frequency information of EEG data in analysis. In this study, we selected eLORETA-ICA which has many advantages over the other network visualization methods as we explained above and applied it to EEG data to obtain complete set of EEG-RS-independent-Ns across several frequency bands for the first time.

## Methods

### Subjects

We recruited 306 healthy elderly subjects who had no history of neurological or psychiatric disorders. Elderly subjects over 60 years old underwent clinical tests to ensure that memory and other cognitive functions were within normal limits (MMSE > 24, CDR = 0). From the participants, 146 subjects were healthy volunteers, and the remaining 160 subjects were ascertained from an epidemiological study among inhabitants in Tone, Ibaraki, Japan. This study was approved by the Ethics Committee of Osaka University Hospital and followed the Declaration of Helsinki. Written informed consent was obtained from the subjects.

### EEG recording and data acquisition

Subjects underwent EEG recordings in a resting state, eyes closed condition for about 5 min. Subjects were instructed to keep their eyes closed while staying awake during the recordings. Spontaneous cortical electrical activity was recorded with a 19-channel EEG system (EEG-1000/EEG-1200, Nihon Kohden, Inc., Tokyo, Japan), filtered through a 0.53–120 Hz band-pass filter, and sampled at 500 Hz. EEG was recorded with the electrodes positioned according to the International 10–20 system (i.e., Fp1, Fp2, F3, F4, C3, C4, P3, P4, O1, O2, F7, F8, T3, T4, T5, T6, Fz, Cz, Pz) using a linked ears reference. Electrode impedances were kept below 5 kΩ. For each subject, 120-s artifact-free, resting-awake segments were manually selected by visual inspection using Neuroworkbench software (Nihon Kohden, Inc., Tokyo, Japan).

### EEG-source reconstruction method

We used eLORETA (exact low resolution brain electromagnetic tomography) to compute the cortical electrical distribution from the scalp electrical potentials measured at the electrode sites (Pascual-Marqui et al., 2011). The eLORETA method is a weighted minimum norm inverse solution, where the weights are unique and endow the inverse solution with the property of exact localization for any point source in the brain. Thus, due to the principles of linearity and superposition, any arbitrary distribution will be correctly localized, albeit with low spatial resolution. In the current eLORETA version, the solution space consists of 6239 cortical gray matter voxels at 5 mm spatial resolution, in a realistic head model (Fuchs et al., 2002), using the MNI152 template (Mazziotta et al., 2001). The LORETA method has been previously used and validated with real human data during diverse sensory stimulation and in neuropsychiatric patients (Dierks et al., 2000; Vitacco et al., 2002; Pascual-Marqui et al., 2011; Aoki et al., 2013a). A further property of eLORETA is that it has correct localization even in the presence of structured noise (Pascual-Marqui et al., 2011). In this sense, eLORETA is an improvement over previously related versions of LORETA (Pascual-Marqui et al., 1994) and sLORETA (Pascual-Marqui, 2002). eLORETA images of spectral density were computed for five frequency bands: delta (2–4 Hz), theta (4–8 Hz), alpha (8–13 Hz), beta (13–30 Hz), and gamma (30–60 Hz) (Canuet et al., 2012).

### Functional ICA

In most of the resting state network (RSN) literature, ICA is the method most widely used for the discovery of sets of regions that work together as networks. There are numerous different processing strategies that are being used in the RS-independent-N literature, as reviewed by Calhoun (Calhoun et al., 2009). For instance, in typical fMRI group studies for the discovery of RS-independent-Ns, the time series images for each subject are first heavily pre-processed (see Calhoun et al., 2009 for details), and then all subjects’ time series images are concatenated. This produces a matrix, where one dimension consists of “space” (i.e., the brain voxels), and the other dimension consists of time. Finally, an ICA algorithm is applied to this matrix, which will produce a set of spatial components (i.e., images), where each “component image” consists of a so-called “network”. In order to interpret a network image, one must threshold its values appropriately, displaying the brain regions that have highest loadings. This post-processing is achieved by z-transforming the component network image, and using an empirical threshold, as in for example (McKeown et al., 1998; Calhoun et al., 2004; Kelly et al., 2010; Agcaoglu et al., 2014). In this way, each network image will display areas whose activities are tightly linked (i.e., they work together as a network).

In contrast to relatively slow hemodynamic images, high time resolution images of electrical neuronal activity can be computed using eLORETA applied to EEG recordings. In an implicit manner, these images contain an additional dimension of frequency. Whereas fMRI images have their spectrum concentrated below 0.1 Hz, EEG contain a wealth of differential functional information in the classical range from 2 to 60 Hz. In order to take into account this additional dimension of information, the classical ICA as applied in fMRI was generalized. All the technical details can be found in Pascual-Marqui and Biscay-Lirio (2011).

For the sake of completeness, a brief description follows. The EEG recording of each subject is first transformed to the frequency domain, using the discrete Fourier transform. This will produce a set of cross-spectral EEG matrices, for each frequency of interest, such as those described above. This information is then used for calculating the spectral density for each cortical voxel and for each frequency band, using the methodology described in detail in Frei et al. (2001). With this initial procedure, each subject contributes five eLORETA images of cortical spectral density (one for each frequency band: delta, theta, alpha, beta, and gamma). From the point of view of mathematics, these data correspond to a “function” of space (cortical voxel) and frequency. In the next step, the data from each subject is concatenated, thus producing a matrix where one dimension corresponds to the different subjects, and the other dimension corresponds jointly to space-frequency. This approach is common in a relatively young field of statistics known as functional data analysis (Ramsay and Silverman, 2005). When ICs analysis is applied to this matrix, a more general form of networks are discovered, and the method is described as functional ICA, given its origin in the field of functional data analysis. Each functional network consists of a set of five images, one for each frequency, because space and frequency and all their possible interactions are now jointly expressed. In contrast to a classical fMRI network image which corresponds to brain regions that “work” together over time, an EEG-eLORETA based functional network corresponds to brain regions and frequencies that “work” together across a population of subjects. This allows not only for the discovery of regions that work together, but also for the discovery of cross-frequency coupling.

In this paper, the number of ICs (networks) is estimated by sphericity test (Bartlett, 1954). In the eLORETA-ICA algorithm, ICs were obtained by maximizing the independence between components which was measured by non-Gaussianity. In particular, non-Gaussianity was calculated by fourth-order cumulant (Cardoso, 1989; Cichocki and Amari, 2002). Then, ICs were ranked according to total EEG power and color coded with a z-score threshold of 3.0, in complete analogy to the methods used in practice in fMRI-ICA networks (as explained in detail above). In the color–coded maps, red and blue colors represent power increase and decrease with increasing IC coefficient which indicates activity of IC, respectively.

## Results

**Figure 1:**
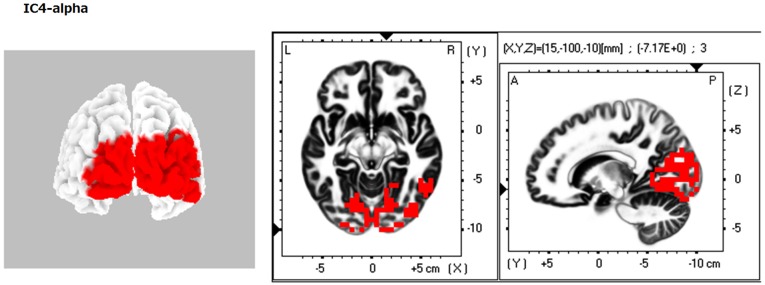
**eLORETA-ICA component 4 (IC4)**. IC4 corresponds to the occipital visual network in alpha frequency band. In the color–coded maps, red and blue colors represent power increase and decrease with increasing IC coefficient, respectively.

**Figure 2:**
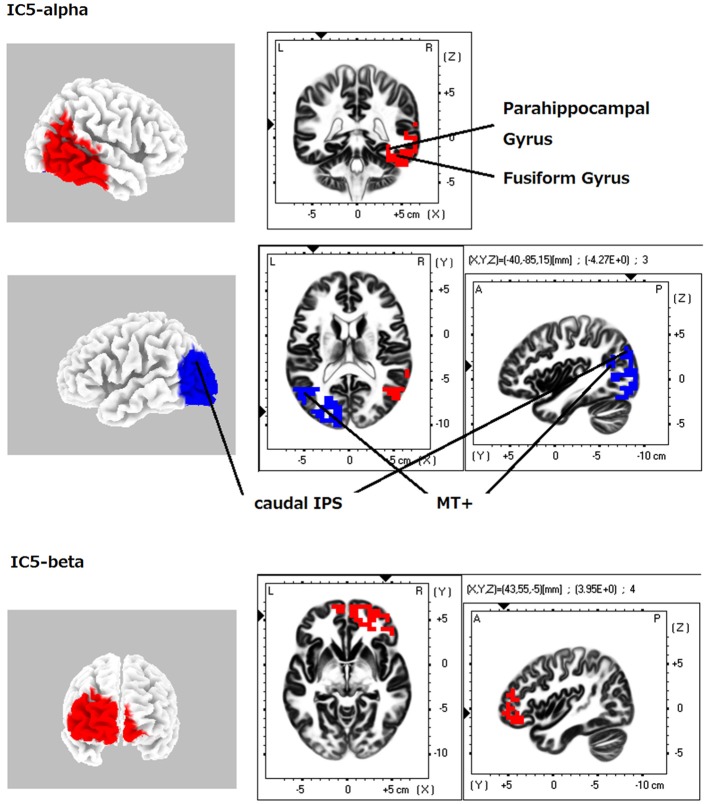
**eLORETA-ICA component 5 (IC5)**. Left IC5 regions (the left posterior occipito-parietal cortex, caudal intraparietal sulcus (caudal IPS) and middle temporal + (MT+)) corresponds to left posterior dorsal visual pathway (DVP). Right IC5 regions (the right occipitotemporal cortex, temporoparietal junction (TPJ), parahippocampal gyrus, fusiform gyrus and ventral prefrontal cortex (vPFC)) corresponds to right ventral visual pathway (VVP). The right VVP links right occipitotemporal cortex in alpha frequency band to the right vPFC in beta frequency band. The left posterior DVP correlates negatively with the areas of the right VVP.

**Figure 3:**
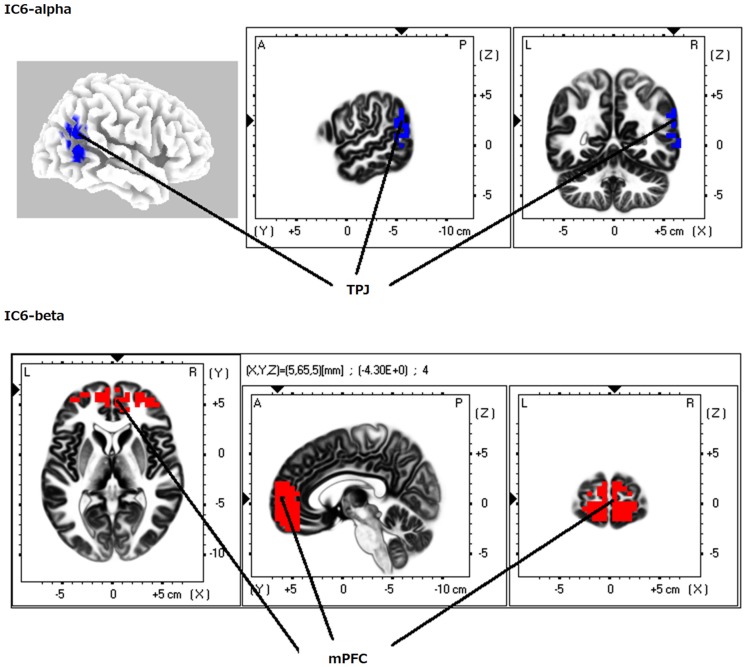
**eLORETA-ICA component 6 (IC6)**. IC6 is formed by the medial PFC (mPFC) in beta frequency band and the right TPJ in alpha frequency band, which shows negative correlation.

**Figure 4:**
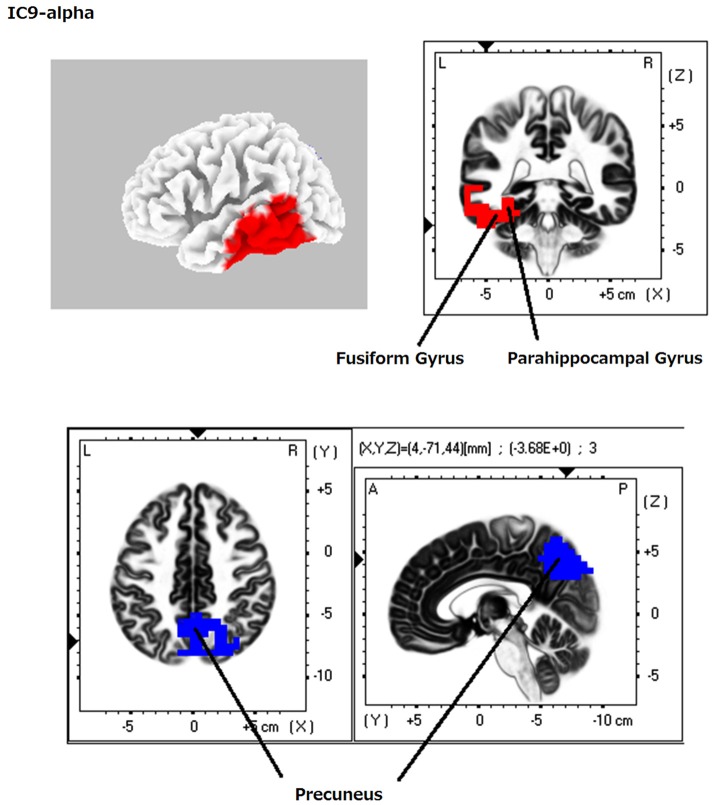
**eLORETA-ICA component 9 (IC9)**. IC9 comprises the precuneus in alpha frequency band and the left VVP in alpha frequency band, which shows negative correlation.

**Figure 5:**
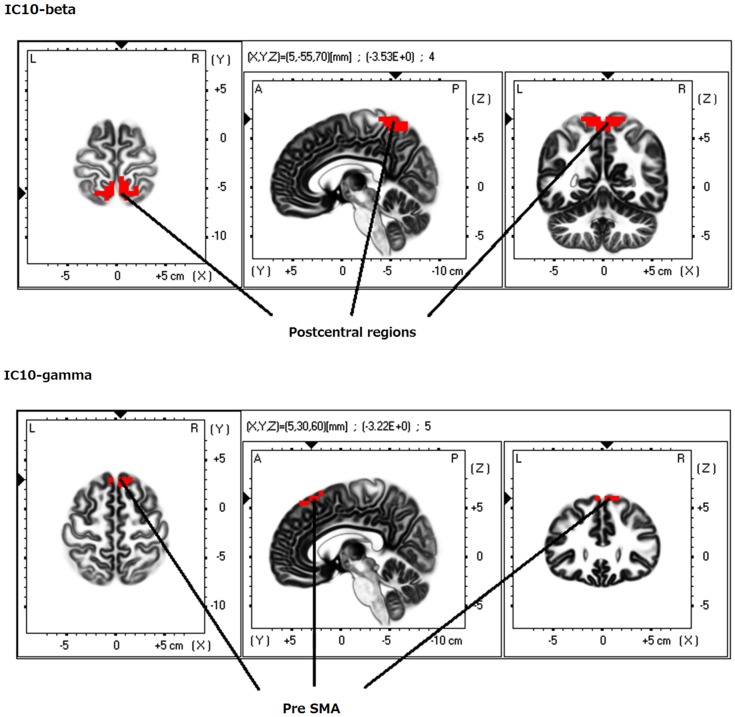
**eLORETA-ICA component 10 (IC10)**. IC10 comprises the medial postcentral regions (Brodmann area 5 and 7) in beta frequency band and the pre supplementary motor area (pre-SMA) in gamma frequency band, which shows positive correlation.

**Figure 6:**
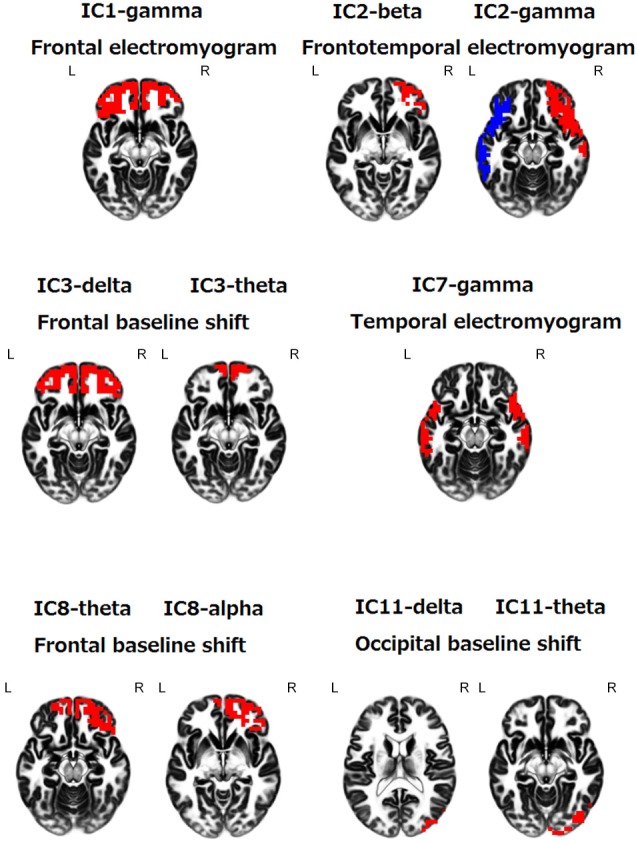
**eLORETA-ICA component 1, 2, 3, 7, 8 and 11 in above written frequency bands**. These components correspond to artifacts of electromyogram or baseline shifts, based on spatial distributions of power and frequency ranges.

Artifact-free 120-s epochs were obtained in 80 out of 306 healthy subjects. The age distribution of the 80 healthy subjects (57 men and 23 women) was as follows: 18–29 years (25 men and 2 women), 30–49 years (15 men and 4 women), 50–69 years (14 men and 11 women) and 70–87 years (3 men and 6 women) (44 ± 20 (mean ± standard deviation)). The median of MMSE scores over 60 years old was 30 (interquartile range; 29–30). It can be seen an overall male predominance, which may reflect a bias of our healthy volunteers, and the female predominance in the 70–87 years group, which may reflect a delay of age-related cognitive decline in female. The number of ICs estimated by the sphericity test was 12.0. Subsequently, we applied eLORETA-ICA as the number of components varied from 11 to 13. Then, 11 ICs were most consistent with physiological assumption that is topography and frequency of some known networks and artifacts such as electromyogram is at frontal or temporal cortex in gamma frequency band, therefore we selected 11 as the number of components. Next, we identified, based on spatial distributions of power and frequency ranges, IC4, IC5, IC6, IC9 and IC10 as RS-independent-Ns (Figures 1–5); IC1, IC2, IC3, IC7, IC8 and IC11 as artifacts of frontal and temporal electromyogram or frontal and occipital baseline shifts (Figure 6).

When identifying the different ICs derived from our analyses, we found that IC4 corresponded to the occipital visual network in alpha frequency band (Figure 1). IC5 consisted of the right VVP, corresponding to the right occipitotemporal cortex and the right ventral prefrontal cortex (vPFC), and the left posterior DVP. The right VVP linked right occipitotemporal cortex in alpha frequency band to the right vPFC in beta frequency band. The left posterior DVP, comprised the ipsilateral posterior occipito-parietal cortex, caudal intraparietal sulcus (cIPS) and posterior end of middle temporal gyrus (MT+) in alpha frequency band, which correlated negatively with the areas of the right VVP (Figure 2). IC6 was formed by the medial PFC (mPFC) in beta frequency band and the right temporoparietal junction (TPJ) in alpha frequency band, which showed negative correlation (Figure 3). IC9 comprised the precuneus in alpha frequency band and the left VVP in alpha frequency band, which also showed negative correlation (Figure 4). IC10 comprised the medial postcentral regions (Brodmann area 5 and 7 (BA 5–7)) in beta frequency band and the pre supplementary motor area (pre-SMA) in gamma frequency band, which showed positive correlation (Figure 5).

## Discussion

In this study, using eLORETA-ICA, we could identify five RS-independent-Ns corresponding to (1) the occipital visual network in alpha frequency band (IC4), (2) the right VVP in alpha and beta frequency bands and left posterior DVP in alpha frequency band (IC5), (3) the mPFC in beta frequency band and right TPJ in alpha frequency band (IC6), (4) the precuneus and left VVP in alpha frequency band (IC9); and (5) the medial postcentral regions in beta frequency band and the pre-SMA in gamma frequency band (IC10).

### Independent component 4

IC4 was found at the occipital cortex in alpha frequency band (Figure 1). It is well known that the occipital cortex is involved in visual perception processing. Consistent with this fact and with our result, previous neurophysiological studies found that visual processing related activity in the occipital regions occurred in the alpha frequency band. In particular, alpha oscillation in the occipital regions is enhanced during no expectation of visual stimulus and is reduced during expectation and presentation of visual stimulus (Klimesch et al., 1998).

### Independent component 5

IC5 was found at the right occipitotemporal cortex in alpha frequency band and at the right vPFC in beta frequency band with left posterior occipito-parietal cortex, cIPS and MT+ in alpha frequency band (Figure 2). Left IC5 regions (the left posterior occipito-parietal cortex, cIPS and MT+) corresponds to left posterior DVP and right IC5 regions (the right occipitotemporal cortex, TPJ, parahippocampal gyrus, fusiform gyrus and vPFC) corresponds to right VVP. DVP is a functional network involved in automatic visual guidance of spatial movements. Within this network cIPS and MT+ is linked to action-relevant features of objects such as shape and orientation from visual information processed in the occipital lobe. Right VVP is a visual recognition network where visual information that has flowed from the occipital lobe is compared to visual/spatial memory in right temporal cortex then identified in right temporal cortex or right vPFC (Fairhall and Ishai, 2007; Kravitz et al., 2011, 2013; Milner, 2012). Taking into account these findings, IC5 corresponds to a network that activity of the right VVP correlated negatively with left posterior DVP activity. Previous accumulating studies revealed that function of DVP is“online” “unconsciously occurred (automatic)” visual perception of spatial components to guide spatial movements, while function of VVP is “off-line” “conscious” visual perception and recognition of feature components (Kravitz et al., 2011, 2013; Harvey and Rossit, 2012; Milner, 2012). Therefore, we can assume IC5 as dual-process of visual perception: the left posterior DVP for automatic visual perception to guide spatial movements and right VVP for detailed perception and recognition of visual input. Our result of negative correlation between right VVP and left posterior DVP is consistent with dual-process of visual perception. In addition, our result of emergence of VVP only on the right side also fit with the fact that right dominant engagement of VVP in visuospatial search and recognition (Corbetta et al., 2005). This negative correlation was also seen in visuospatial neglect patients, who injured right VVP area, enhanced left posterior DVP activity (not whole DVP) at acute stage and attenuated its activity with clinical recovery (Corbetta et al., 2005; He et al., 2007; Rossit et al., 2012).

### Independent component 6

IC6 was found at the mPFC in beta frequency band and right TPJ in alpha frequency band (Figure 3). Medial PFC is anterior hub of the DMN and right TPJ is a hub of the right VAN (Corbetta and Shulman, 2002; Buckner et al., 2008). Connectivity analysis of resting fMRI data has showed that mPFC has maximal positive connectivity with right posterior TPJ (Mars et al., 2012; Kubit and Jack, 2013). Taking into account these findings, IC6 corresponds to a network that activity of anterior hub of DMN (mPFC) positively correlated with that of right VVP. The DMN enhance its activity in autobiographical memory retrieval (Cabeza et al., 2004). However autobiographical memory retrieval involves both self-referential processing and memory retrieval process. So, Kim (2012), by subtracting fMRI activity in laboratory-based memory retrieval from autobiographical memory retrieval, found that self-referential processing was related to mPFC, right parahippocampal cortex and posterior cingulate cortex (PCC). So, we can speculate IC6 as self-referential processing. In support to this notion, there is a case report of a patient with loss of the sense of self-ownership who also showed hypometabolism in the right inferior temporal cortex as well as in the right parietooccipital junction and precentral cortex (Zahn et al., 2008).

### Independent component 9

IC9 was found at the precuneus and left VVP in alpha frequency band (Figure 4). The precuneus is dominantly related to familiarity of the memory (Yonelinas et al., 2005) and left VVP is memory recognition area whose activation reflects retrieval and identification of memory (Cabeza, 2008; Ravizza et al., 2011; Angel et al., 2013). IC9 showed the precuneus was negatively correlated with left VVP in alpha frequency band. Consistent with our result, EEG study using sLORETA showed the same correlation between decreasing alpha power in the precuneus and increasing alpha power in the left temporal cortex with WM load during WM retention period in some healthy subjects (Michels et al., 2008). Dual-process models of memory recognition have been proposed by many researchers which suggest memory has two separate systems: familiarity of the memory (sense of knowing) and recollection (Yonelinas, 2002). In memory retrieval, the precuneus engages in familiarity, while left VVP regions (left TPJ, parahippocampal cortex and hippocampal formation) engage in episodic memory retrieval (Yonelinas et al., 2005; Sestieri et al., 2011). Familiarity is a working memory which is a sense of knowing temporarily occurred (several tens of seconds) after encoding. That is, familiarity is “unconsciously occurred (automatic)” “online” “sensory component” of short-term memory to be manipulated in multiple cognitive processes (working memory). On the other hand, episodic memory retrieval is a “conscious” “off-line” “detailed” perception and recognition of long-term episodic memory (Baddeley and Hitch, 1974; Huijbers et al., 2010, 2012). Therefore, we can conclude that familiarity and episodic memory have properties of the DVP and the VVP, respectively (please refer to the discussion of IC5). In fact, the precuneus showed strong coherence with DVP by fMRI connectivity analysis (Huijbers et al., 2012). Taken together, we can speculate that IC9 reflects dual-process of memory perception: the precuneus for automatic sensory component of the memory to guide multiple cognitive processes in memory domain and left VVP for detailed perception and recognition of episodic memory. Our results elucidated that similarity of perception and recognition between vision (IC5) and memory (IC9). Lesion studies also presented a case of neglect in memory domain analogous to visuospatial neglect: patients who had bilateral TPJ lesions showed a deficit in detailed memory retrieval in free recall (subserved by the left VVP), although they can access to these memories when guided by probe questions (function subserved by the precuneus; Berryhill et al., 2007; Cabeza, 2008).

### Independent component 10

IC10 was found at the medial postcentral regions (BA5–7) in beta frequency band, and at the pre-SMA in gamma frequency band (Figure 5). Beta activity in medial sensory regions is known as Rolandic beta rhythm, which is typically observed in resting state and suppressed by voluntary movements (Pfurtscheller, 1981). This beta oscillation is thought as an idling rhythm of sensory regions (Ritter et al., 2009). From our result, gamma oscillation in the pre-SMA can also be assumed as idling rhythm of pre-SMA. In support of this notion, the gamma oscillation in the pre-SMA was suppressed by voluntary movements (Hosaka et al., 2014). Taking into account these findings, we identified IC10 as sensorimotor network.

Overall, topographies of alpha and beta frequency bands is consistent with their roles: alpha oscillation for inhibition of the visual pathway (Snyder and Foxe, 2010; Capotosto et al., 2012; Capilla et al., 2014), beta oscillation in PFC for higher cognitive functions such as evaluation and prediction (Arnal et al., 2011; Hanslmayr et al., 2011; Buschman et al., 2012; Aoki et al., 2013b; Kawasaki and Yamaguchi, 2013) and beta oscillation in sensorimotor area for motor control (Engel and Fries, 2010).

This is the first study presenting ICs using eLORETA-ICA with resting state EEG data, and more importantly, which highlight the differences in some aspects from the previous RS-independent-Ns using ICA with resting state fMRI data. First, eLORETA-ICA of EEG data presented right and left VVP separately, strikingly different from ICA results of fMRI data showing VVP bilaterally. However, de Pasquale et al. (2010) using correlation analysis showed that MEG has greater correlations between intra-hemispheric nodes than inter-hemispheric nodes in RSNs. They elucidated that this difference stemmed from the difference of temporal resolution: EEG and MEG have much higher temporal resolution (1–2 ms) of cortical activity than fMRI, which has 2 s temporal resolution. These findings indicate that only EEG and MEG, which have millisecond temporal resolution, combined with ICA can detect the correct ICs of cortical activity. Furthermore, our result of right and left separation of VVP is consistent with previous findings that left lateralized activation of VVP during episodic memory retrieval and right lateralized activation of VVP during visual target detection (Corbetta et al., 2005; Daselaar et al., 2006; Angel et al., 2013). Second, our results were restricted to cortical areas whereas RSNs derived from fMRI data included deep brain structures such as basal ganglia, hippocampus and cingulate cortex. This caused from the fact that EEG cannot detect electrical activity of the deep brain because electrical potential drastically attenuated by conduction from deep brain to the surface of the head. Therefore, for instance, we cannot determine the PCC is involved in IC6 or IC9, although controversy exists whether the PCC should be involved in self-referential processing or episodic memory retrieval (Kim, 2012; Angel et al., 2013).

Although the fact is known that cortical electrical activity reconstructed from EEG data using sLORETA showed several topographic distributions somewhat similar to RS-independent-Ns for a short period (microstate; Musso et al., 2010), no one could extracted independent sets of cortical electrical activity (EEG-RS-independent-Ns). And there are some other decomposing methods such as principal component analysis and correlation analysis, they cannot decompose cortical electrical activity into ICs in a precise sense because cortical electrical activity is non-Gaussian (Bell and Sejnowski, 1997; Hyvärinen and Oja, 2000; Stam, 2005; Mantini et al., 2011). Therefore, we selected eLORETA-ICA to detect EEG-RS-independent-Ns.

Our results should be interpreted with caution based on the following limitations. First, relative small number of electrodes (19 electrodes) and realistic head model in eLORETA may affect the source localization results. However, the good localization property of the LORETA tomography was validated in several studies as we mentioned in the Methods section and our source localization results of eLORETA-ICA are consistent with neuroimaging findings of RSNs. Second, low spatial resolution of eLORETA, which blur the cortical sources, may cause non-detection of the low-electrical-activity cortical sources. Thus, subsequent ICA may have missed some low activity RS-independent-Ns. Third, our present study has made use of the hypothesis that healthy subjects have common RS-independent-Ns which are consistent throughout a very wide age range, thus aging-related changes are restricted to activities of RS-independent-Ns (IC coefficients). However, we confirmed that occipital basic oscillations of all subjects were in the alpha frequency band by visual inspection and almost all the RS-independent-N results did not change even excluding 9 subjects aged 70 years or more from eLORETA-ICA. In addition, our source localization results of eLORETA-ICA are consistent with neuroimaging findings of RSNs. Fourth, we supposed correspondences between RS-independent-Ns and functional networks. However, these correspondences should be confirmed by comparing the activities of RS-independent-Ns with cognitive test scores in the future study.

## Conclusion

We selected eLORETA-ICA which has many advantages over the other network visualization methods and overall findings indicate that eLORETA-ICA with EEG data can identify five RS-independent-Ns with their intrinsic oscillatory activities, as well as functional correlations within these networks, while conventional methods used to examine RSNs such as fMRI with functional tasks or fMRI with ICA have not been shown to do so. Moreover, once RS-independent-Ns are determined by eLORETA-ICA, this method can accurately identify activity of each RS-independent-N from EEG data as it removes EEG artifacts by separating artifact components. Therefore, eLORETA-ICA with EEG data may represent a useful and powerful tool to assess activities of RS-independent-Ns, which correspond to specific functions, in patients with neuropsychiatric disease such as dementia and depression.

## Conflict of interest statement

The authors declare that the research was conducted in the absence of any commercial or financial relationships that could be construed as a potential conflict of interest.
